# Hepatoprotective and immunomodulatory effects of copper-nicotinate complex against fatty liver in rat model

**DOI:** 10.14202/vetworld.2019.1903-1910

**Published:** 2019-12-04

**Authors:** Ahmed Medhat Hegazy, Ayman Samir Farid, Ahmed S. Hafez, Rania M. Eid, Soad M. Nasr

**Affiliations:** 1 Department of Forensic Medicine and Toxicology, Faculty of Veterinary Medicine, Aswan University, Sahari, Airport Way 81528, Aswan, Egypt; 2 Department of Clinical Pathology, Faculty of Veterinary Medicine, Benha University, Moshtohor, Toukh 13736, Qalyubia, Egypt; 3 Department of Pharmacology, Faculty of Veterinary Medicine, Aswan University, Sahari, Airport Way 81528, Aswan, Egypt; 4 Department of Physiology, Faculty of Medicine, Aswan University, Sahari, Airport Way 81528, Aswan, Egypt; 5 Department of Parasitology and Animal Diseases, National Research Centre, 33 Bohouth Street, Post Box 12622, Dokki, Giza, Egypt

**Keywords:** copper-nicotinate complex, cytokines gene expression, fatty liver, hepatoprotective, oxidative stress markers

## Abstract

**Aim:**

The current study was designed to evaluate the potential hepatoprotective and immunomodulatory effects of copper-nicotinate complex (CNC) against methionine- and choline-deficient diet (MCDD)-induced fatty liver in rats.

**Materials and Methods:**

Forty male Wistar rats were randomly allocated into one of four equal-sized groups (G1-G4). The G1 group was fed a balanced diet and kept under normal conditions; the G2 group received CNC orally at a dose of 0.043 mg/kg body weight, 3 times/week for 4 weeks, and a balanced diet; the G3 group was fed an MCDD for 4 weeks; and the G4 group was fed an MCDD and administered CNC at the same dose and route as G2. Blood samples were collected for the determination of serum enzyme activity. After 4 weeks of treatment, liver specimens were collected for the evaluation of the oxidative/antioxidative markers, cytokine gene expression, and histopathological examination.

**Results:**

CNC improved MCDD-induced liver dysfunctions by recovering serum alanine aminotransferase, aspartate aminotransferase, and gamma-glutamyl transferase activities to their normal levels. The glutathione (GSH) level and superoxide dismutase (SOD) activity significantly decreased, while lipid peroxidation (as reflected by malondialdehyde [MDA]) markedly increased in the liver tissue of the MCDD group. After cotreatment with MCDD and CNC, the GSH level and SOD activity markedly increased and the MDA level significantly decreased to return to normal levels. After cotreatment with MCDD and CNC, significant downregulation of the mRNA expression of hepatic interleukin (IL)-1β, IL-4, macrophage inflammatory protein-1α, and monocyte chemoattractant protein-1 genes was found. Moreover, CNC reduced fatty liver complications by reducing the number of hepatic vacuolations, degenerative changes in the hepatocytes, and hemorrhage.

**Conclusion:**

CNC has the potential to limit tissue injury and possibly prevent the progression to severe liver disease caused by an MCDD.

## Introduction

Non-alcoholic fatty liver disease (NAFLD) is the most common chronic liver disorder across the world. It has a prevalence of approximately 20% in the general population and up to 95% in people with obesity. This liver disease includes mild-to-severe steatosis, steatohepatitis, hepatocellular injury, progressive chronic inflammation, and fibrosis [1]. NAFLD occurs due to morphologic and functional alterations of the mitochondria, an increase of inflammatory activity, oxidative stress, generation of reactive oxygen species, and lipid peroxidation [2]. Metabolic disorders such as insulin resistance, dyslipidemia, and visceral obesity are the main risk factors for NAFLD.

A methionine- and choline-deficient diet (MCDD) is used in rodents to induce NAFLD. Choline is a nutrient that influences lipid metabolism [3] through lipid second messengers [4], methylation-dependent biosynthesis of molecules including those in regulation of gene expression [5], activation of nuclear receptors [6], enterohepatic circulation of bile and cholesterol [7], plasma membrane fluidity [8], and mitochondrial bioenergetics [9]. Methionine is essential for the growth, repair, and metabolism of all tissues. In the diet, the methionine level is important in relation to the need for choline as a methyl donor [10]. Nutritional models based on MCDD lead to the impaired formation of very low-density lipoproteins, inducing pathogenic events such as the early accumulation of triglycerides in the liver (fatty liver) with increased lipid peroxidation followed by inflammation and hepatic fibrosis [11]. Copper is an essential trace element. It is a cofactor for many enzymes that are essential for respiration, bone formation, and development of connective tissue, and an essential catalytic cofactor of some metalloenzymes [12]. Deficiency of certain vitamins, such as nicotinic acid, has deleterious effects [13]. Copper-nicotinate complex (CNC) is a biologically active copper-chelating complex that exhibits superoxide dismutase (SOD)-mimicking activity, anti-inflammatory action in gastric ulcer [14], and reduction of hepatocellular carcinoma [15], acts a rheumatoid arthritis treatment [16], improves skin burns [17], ameliorates nephrotoxicity resulting from the topical application of glycerol [14], and protects against neuronal and glial cell injuries [18].

Therefore, this study was conducted to evaluate the potential hepatoprotective and immunomodulatory effects of CNC in Wistar rats exposed to MCDD-induced fatty liver.

## Materials and Methods

### Ethical approval

This experiment was carried out according to the guidelines of the Institutional Animal Ethics Committee, Benha University, Egypt, and Approval Protocol No.: 44 on April 10, 2016.

### CNC

CNC was provided as a gift by Professor Dr. Ahmed Yassen Nassar, Professor of Biochemistry, Faculty of Science, Assiut University, Egypt. The complex was administered to rats at a dose of 0.043 mg/kg body weight, 3 times/week, continuously for 4 weeks, according to a previously described method [14].

### Composition of diets

The balanced diet composition (Table-1) was formulated according to the National Research Council (NRC) [19]. It contained crude protein (15%), metabolizable energy (ME) (3800 kcal/kg diet), choline (1342 ppm), and methionine (0.32%), as shown in Table-2.

**Table-1:** Ingredients of the balanced diet.

Feed ingredients	Diet (%)
Sunflower oil	15.000
Concentrate mixture (45%)	10.000
Yellow corn	49.000
Soybean meal (44%)	11.000
Wheat bran	10.000
Molasses	03.000
Common salt	00.500
Ground limestone	00.200
Dicalcium phosphate	00.100
Lysine	00.200
DL-methionine	00.700
Mineral-vitamin premix	00.300
Total	100.000

**Table-2:** Chemical constituents of the balanced and MCDD.

Item	Balanced diet	MCDD
Crude protein (%)	15.000	15.000
Metabolizable energy (kcal/kg diet)	3800	3800
Methionine (%)	0.320	0.240
Choline (ppm)	1342	575

MCDD=Methionine- and choline-deficient diet

The MCDD (Table-3) was formulated according to the NRC [19]. It contained crude protein (15%), ME (3800 kcal/kg diet), choline (575 ppm), and methionine (0.24%), as shown in Table-2.

**Table-3:** Ingredients of the methionine- and choline-deficient diet.

Feed ingredients	Diet (%)
Corn	58.275
Soya 46%	21.900
Oil	15.000
Limestone	1.200
Sucrose	3.000
Lysine	0.200
Common salts	0.125
Premix choline free	0.300
Total	100.000

### Experimental animals

Five-week-old male Wistar rats (120-140 g) were obtained from the Animal House, National Cancer Institute, Cairo, Egypt. All animals were housed in clean cages and given food and water *ad libitum*. Their environmental conditions were controlled in terms of light (12 h light-dark cycle starting at 8:00 a.m.) and room temperature (23±3°C).

### Experimental design

Forty male Wistar rats were randomly allocated into one of four groups (10 rats per group). The first group (G1) was kept as the control and fed a balanced diet. The second group (G2) was given CNC at a dose of 0.043 mg/kg body weight orally through a stomach tube 3 times/week continuously for 4 weeks and fed a balanced diet. The third group (G3) was fed an MCDD for 4 weeks continuously. The fourth group (G4) was fed an MCDD continuously for 4 weeks and given CNC orally through a stomach tube (0.043 mg/kg body weight) 3 times/week continuously for 4 weeks. Clinical signs were recorded for all groups during the experimental period. At the end of the 4^th^ week of the experiment, blood samples were collected from the retro-orbital venous plexus of each rat. Each blood sample was placed in a plain centrifuge tube for the separation of the serum. The serum samples were stored at −20°C for further enzyme analyses. At the end of the 4^th^ week of the experiment, the rats were euthanized. Three portions of liver specimens were collected from all rats in each group. The first and second portions of the liver specimen were stored at −70°C for further evaluation of the oxidative/antioxidative markers and cytokine gene expression. The third portion of the liver specimens was collected and fixed in 10% formal saline for histopathological examinations.

### Preparation of liver homogenates

Preparation of liver tissue homogenates was performed according to a previously published study [20]. Liver homogenate (prepared from 1 g of hepatic tissue) was obtained from all rats at the end of the 4^th^ week of the experiment, washed, and homogenized in an ice-cold 1.15% solution of potassium chloride in 50 mmol potassium phosphate buffer solution (pH 7.4) to obtain a 10% liver homogenate (W/V; weight of liver tissue [g] per volume of the buffer [ml]). Homogenization of the liver tissue was performed using a Sonicator (4710 Ultrasonics Homogenizer, Cole-Parmer Instrument Co., USA). The homogenate was then centrifuged (Sigma 30K refrigerated centrifuge; Sigma-Aldrich Co., St. Louis, MO, USA) at 4000 rpm for 5 min at 4°C. The collected supernatant was used in the determination of the concentration of reduced glutathione (GSH) and lipid peroxidation byproducts and determination of the SOD activity.

### Assay methods

#### Determination of serum liver enzymes

Colorimetric determination of the alanine aminotransferase (ALT) and aspartate aminotransferase (AST) activity was performed according to a previously described method [21]. The activity of gamma-glutamyl transferase (GGT) was also assessed according to a previously published method [22].

#### Determination of oxidative/antioxidative markers in liver homogenates

The reduced GSH content was measured according to the method described by Ellman [23]. The reduced chromogen is directly proportional to the GSH concentration and its absorbance was measured at 405 nm using a spectrophotometer (Model, JASCO 7800, UV/VIS, Japan). Concentrations of GSH are expressed as µmol/mg protein. SOD activity was measured according to the method described by Misra and Fridovich [24]. The enzyme activity was determined by considering the degree of inhibition of the autoxidation of epinephrine in alkaline medium. SOD activity is expressed as unit/mg protein. Lipid peroxidation byproducts in liver tissue homogenates were determined according to the method described by Ohkawa *et al*. [25], which depend on the formation of malondialdehyde (MDA) as an end product of lipid peroxidation that reacts with thiobarbituric acid-producing thiobarbituric acid reactive substance, a pink chromogen that can be measured spectrophotometrically at 535 nm. An MDA standard was used to construct a standard curve against which readings of the samples were plotted. Concentrations of lipid peroxides are expressed as nmol/mg protein. The total protein content of the homogenates was determined using the method described by Lowry *et al*. [26].

Analysis of mRNA expression of hepatic interleukin (IL)-1β, IL-4, IL-10, monocyte chemoattractant protein (MCP)-1, and macrophage inflammatory protein (MIP)-1α genes using real-time polymerase chain reaction (PCR).

The expression of hepatic IL-1β, IL-4, IL-10, MCP-1, and MIP-1α cytokines was analyzed using real-time PCR with sense and antisense primers throughout the experiment as previously described [27] using the following primers sets: IL-1β (GenBank ID: M98820.1), sense (5′-CAC CTC TCA AGC AGA GCA CAG-3′) and antisense (5′-GGG TTC CAT GGT GAA GTC AAC-3′); IL-4, sense (5′-CAG GGT GCT TCG CAA ATT TTA C-3′) and antisense (5′- ACCG AGA ACC CCA GAC TTG TT-3′); IL-10 (GenBank ID: L02926.1), sense (5′-AGA AGC TGA AGA CCC TCT GGA TAC-3′) and antisense (5′-GCT CCA CTG CCT TGC TTT TAT T-3′); MCP-1, sense (5′- ATG CAG TTA ATG CCC CAC TC-3′) and antisense (5′- TTC CTT ATT GGG GTC AGC AC-3′); MIP-1α, sense (5′-CAT TCC TGC CAC CTG CAA AT-3′) and antisense (5′- CAA GTG AAG AGT CCC TGG ATG TG-3′); and 18S rRNA as a housekeeping gene, sense (5′-GAG GTG AAA TTC TTG GAC CGG-3′) and antisense (5′-CGA ACC TCC GAC TTT CGT TCT-3′).

Thermal cycling and fluorescence detection were performed using a 7300 real-time PCR system (Applied Biosystems, Foster City, CA, USA). Changes in gene expression were calculated from the obtained cycle threshold (C_t_) values provided by the real-time PCR instrumentation using the comparative C_t_ method to a reference (housekeeping) gene (18S rRNA) [28].

### Histopathological examinations

Tissue samples were taken from the liver of rats in the different groups and fixed in neutral buffered formalin (10%). Washing was performed using tap water; serial dilutions of alcohol were then used for dehydration. Specimens were cleared in xylene and embedded in paraffin at 56°C in a hot air oven for 24 h. Paraffin beeswax tissue blocks were prepared for sectioning at 4-µm thickness. The obtained tissue sections were collected on glass slides, deparaffinized, and stained with hematoxylin and eosin [29] for histopathological examination using light microscopy.

### Statistical analysis

Statistical analysis was performed using the Statistical Software Package (SPSS) for Windows (Version 20.0; SPSS Inc., Chicago, IL, USA). The significance of differences between the groups was evaluated using one-way analysis of variance followed by Duncan’s multiple range test. Differences were considered significant at p<0.05 level. All data are expressed as mean±standard deviation.

## Results

No mortality was recorded during the period of the experiment.

### Changes in serum liver enzymes

After 4 weeks of treatment, ALT, AST, and GGT activities were significantly (p<0.01) higher in the fatty liver group fed MCDD (G3) compared to those in control (G1) and CNC-treated (G2) groups. On the other hand, when compared to both the control (G1) and CNC alone (G2) groups, the group fed with MCDD and cotreated with CNC (G4) showed recovery of ALT, AST, and GGT levels to normal values (Table-4).

**Table-4:** Effect of CNC on liver enzyme activity in the serum of rats with fatty liver (mean±standard deviation, n=10).

Group parameters	Normal control (G1)	CNC (G2)	MCDD (G3)	CNC+MCDD (G4)
ALT (IU/l)	24.169±1.245^b^	25.966±2.123^b^	48.121±0.924^a^	28.488±2.659^b^
AST (IU/l)	118.944±4.219^b^	124.478±3.651^b^	176.358±9.347^a^	134.604±3.802^b^
GGT (IU/l)	4.892±0.428^b^	4.666±0.462^b^	9.506±0.252^a^	4.756±0.882^b^

Means with different superscripts in the same row are significantly different at p<0.05. CNC=Copper-nicotinate complex, MCDD=Methionine- and choline-deficient diet, ALT=Alanine aminotransferase, AST=Aspartate aminotransferase, GGT=Gamma glutamyl transferase

### Changes in the hepatic oxidative/antioxidative markers

The GSH and SOD activities in the liver tissue of the MCDD-treated group were significantly (p<0.05) lower than those in the control and CNC-treated groups, while the MCDD group cotreated with CNC exhibited recovery of the enzymatic activities to normal levels after the 4^th^ week of the experiment. Likewise, the level of MDA, a marker of lipid peroxidation, in liver tissue was markedly (p<0.01) higher in the MCDD-treated group compared to that in the control and CNC-treated groups, while the MCDD group cotreated with CNC demonstrated recovery of MDA levels to normal values after the 4^th^ week of the experiment (Table-5).

**Table-5:** Effect of CNC on oxidative/antioxidative markers in liver homogenate of rats with fatty liver (mean±standard deviation, n=10).

Group parameters	Normal control (G1)	CNC (G2)	MCDD (G3)	CNC+MCDD (G4)
Glutathione reduced (µmol/mg protein)	16.858±1.777^a^	19.199±4.177^a^	6.003±4.325^b^	13.569±2.621^a^
Superoxide dismutase (unit/mg protein)	0.038±0.008^b^	0.056±0.004^a^	0.007±0.002^c^	0.032±0.006^b^
Malondialdehyde (nmol/mg protein)	10.777±1.384^b^	10.867±2.572^b^	26.671±5.911^a^	14.454±2.102^b^

Means with different superscripts in the same row are significantly different at p<0.05. CNC=Copper-nicotinate complex, MCDD=Methionine- and choline-deficient diet

### Changes in hepatic IL-1β, IL-4, IL-10, MCP-1, and MIP-1α mRNA expression

**Figure-1:**
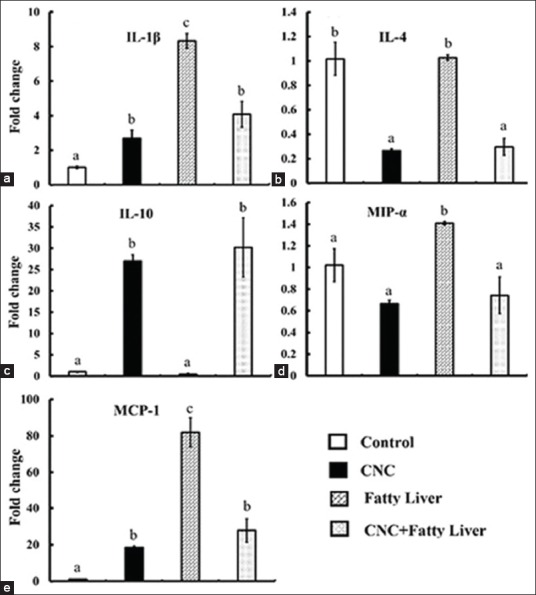
mRNA expression of hepatic interleukin (IL)-1β, IL-4, IL-10, macrophage inflammatory protein-1α, and monocyte chemoattractant protein-1 genes in different experimental groups. Total RNA was prepared from hepatic tissues of each rat group after 4 weeks of treatment. The expression levels were evaluated using real-time polymerase chain reaction. *p<0.05 compared with control values. Bars represent means±standard error (n=4).

To evaluate the molecular mechanisms underlying the immunomodulatory properties of CNC, the mRNA expression of hepatic tissue was investigated (Figure-1). IL-1b, IL-4, MCP-1, and MIP-1α gene expression were significantly upregulated in the fatty liver group (G3) relative to expression in the control group (G1). Meanwhile, there was a non-significant difference in IL-10 expression in the fatty liver group (G3) relative to that in the control group. However, the group cotreated with CNC and MCDD (G4) exhibited significant downregulations in IL-1b, IL-4, MCP-1, and MIP-1α relative to that in the fatty liver group (G3). Both groups that received CNC (G2 and G4) showed significant upregulation in IL-10 expression relative to that in both the control (G1) and fatty liver (G3) groups.

### Histopathological findings

**Figure-2:**
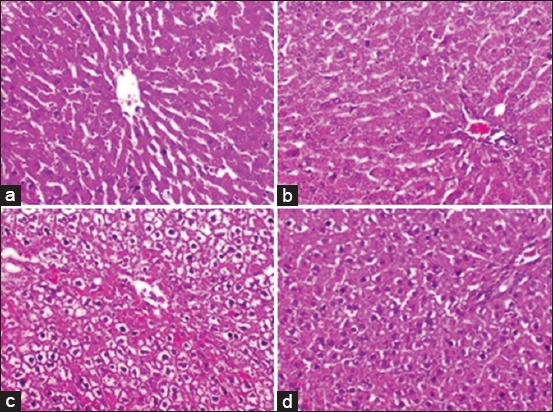
Liver sections of rats. (a) Control group (G1) exhibited normal histological structure. (b) Copper-nicotinate complex (CNC) group (G2) exhibited normal histological structure of the hepatocytes in the hepatic cords with central veins. (c) Methionine- and choline-deficient diet (MCDD) group (G3) exhibited diffuse hepatic vacuolation and centrolobular hepatic degeneration with hemorrhage. (d) CNC with MCDD group (G4) exhibited mild-to-moderate hepatic vacuolation (hematoxylin and eosin, 200×).

The liver of rats in the control group (G1) (Figure-2a) showed normal histological structures. Furthermore, no histopathological alterations were noticed in the liver of rats (G2) that received CNC (Figure-2b). In the fatty liver group fed MCDD (G3), the liver showed defuses hepatic vacuolations and centrilobular hepatic degeneration with hemorrhage. The vacuolation was suspected to be fat accumulation (Figure-2c). However, the liver of rats that received both MCDD (fatty liver) and CNC (G4) appeared normal with mild-to-moderate hepatic vacuolations (Figure-2d).

## Discussion

NAFLD is associated by many with metabolic syndrome [30]. The diagnosis of NAFLD occurs after finding raised aminotransferase levels, fatty liver, or hepatomegaly [31].

In this study, the fatty liver group of rats (G3) exposed to MCDD showed no clinical signs of toxicity. Importantly, there were significant elevations in the ALT, AST, and GGT activities in the serum of the G3 group. Increased levels of these enzymes in the serum may be due to hepatocellular damage induced by the early accumulation of lipid and to a lesser extent on inflammatory processes. Similar results have been previously reported [32].

The main mechanism of MCDD-induced liver steatosis in the rat model is impaired phosphatidylcholine synthesis, which is essential for the secretion of hepatic very low-density lipoproteins [33].

On the other hand, there was a significant decrease in the ALT, AST, and GGT activities in the serum of rats cotreated with MCDD and CNC (G4). This decrease may be due to the hepatoprotective activity of CNC. CNC may act by preventing further oxidative stress (free radical accumulation) in the fatty liver [34]. CNC also enhances the synthesis of SOD or its SOD mimetic activity leads to scavenging of oxygen free radicals. CNC exhibits lipophilic activity that enhances its migration toward the intracellular space of hepatic tissue. It acts as a mediator for cellular copper-dependent enzymes [35].

In the present study, there was an increase in lipid peroxidation, as indicated by an elevation in the MDA level, and decrease in the GSH level and SOD activity in rats that received MCDD after the 4^th^ week of treatment. This might be due to the peroxidation of cell membrane lipids and injury to the cellular components [14]. A reduction in the MDA level and increase in the GSH level and SOD activity were observed with MCDD and CNC cotreatment. CNC is a rich source of antioxidants [36] and seems to be more efficient as detoxifying agent [37]. Its detoxifying action is attributed to its scavenging of free radicals, exhibiting SOD-mimicking activity [14].

Oxidative stress has been recorded in several liver diseases. It also triggers the production of inflammatory cytokines, causing inflammation, and fibrogenic response injury [38]. The presence of inflammation can predict the progression of non-alcoholic steatohepatitis (NASH) to advanced fibrosis [39]. Many cytokines have been suggested to play an essential pathogenic role in NAFLD [40]. In addition, lipid peroxidation end products are potent chemoattractants for inflammatory cells [41] and can activate hepatic stellate cells and hence stimulate hepatic fibrosis [42].

In the present study, we found a significant increase in the expression of hepatic IL-1β in the group with a fatty liver when compared with that in the control group. IL-4 is an anti-inflammatory cytokine. It stimulates lymphocytic differentiation into Th2 cells, contributing to the development of fibrosis and consequent hepatic injury repair [43]. IL-4 is involved in tissue repair and the control of Th1 responses through the alternative activation of macrophages [44]. Thus far, several previous studies have demonstrated that IL-4 may have a pro- or anti-inflammatory role in the development of organ-specific inflammation in classical autoimmune disease models. In experimental immune-mediated hepatitis, a pro-inflammatory role of IL-4 is heavily supported [45]. In this study, the hepatic IL-4 mRNA expression of rats that received CNC (G2 and G4) was very low, while that of IL-10 was high. IL-10 (cytokine synthesis inhibitory factor) is a cross-regulatory factor of Th1 and Th2 responses. It is produced by Th2 cells [46]. In liver tissue, IL-10 may act as an anti-inflammatory mediator against necroinflammatory lesions [47].

The β-chemokines are exclusively chemotactic for mononuclear cells; archetypes of this group, MCP-1 and MIP-1α, are monocyte and lymphocyte chemoattractants [48]. Both TNF-α and IL-1 are potent inducers of MCP-1 [49]. In the liver inflammatory process, MCP-1 may directly cause impairment of hepatocyte proliferation [50]. It regulates the migration of monocytes into tissues and their subsequent differentiation into macrophages [51]. MCP-1 may play an important role in the stimulation of the inflammatory infiltrate. It might also have immunomodulatory effects including enhanced expression of adhesion molecules in monocytes and promotion of a pro-inflammatory cytokine synthesis [52]. In this study, MCP-1 was significantly higher in the fatty liver group (G3) and closer to normal levels in the presence of CNC treatment (G4).

These findings were corroborated by those of the histopathological examination. It revealed the improvement in histopathological features in the liver of CNC-treated rats (G4) compared to the liver of MCDD-treated rats (G3), which showed diffuse hepatic vacuolation, centrilobular hepatic degeneration, and hemorrhage. The vacuolation was suspected to be due to fat accumulation. Mild-to-moderate hepatic vacuolations were noticed in cotreated group (G4). These findings coincide with those of the previous study [53] that fed rats a high carbohydrate, fat-free semi-synthetic diet, and cotreated with CNC.

## Conclusion

This study provides new evidence about the role of CNC as a hepatoprotective and immunomodulatory agent that might represent a novel therapeutic target in NASH with the potential to limit tissue injury and possibly prevent the progression to severe liver disease.

## Authors’ Contributions

AMH designed the study, collected the samples, analyzed the data, and wrote the manuscript. ASF applied PCR testing and revised all manuscripts. ASH obtained the CNC. RME and SMN revised and approved the final manuscript.
